# Creation and Validation of an Algorithm for Predicting the Recurrence of Atrial Fibrillation Following Pulmonary Vein Isolation by Utilizing Real-World Data and Ensemble Modeling Techniques

**DOI:** 10.7759/cureus.43234

**Published:** 2023-08-09

**Authors:** Gaither W Horde, Deepak Ayyala, Paul Maddux, Aaron Gopal, William White, Adam E Berman

**Affiliations:** 1 Department of Medicine, Augusta University Medical College of Georgia, Augusta, USA; 2 Department of Population Health Sciences, Augusta University Medical College of Georgia, Augusta, USA

**Keywords:** catheter ablation, modeling, real-world data, atrial fibrillation, electrophysiology

## Abstract

Introduction

Catheter ablation (CA) of atrial fibrillation (AF) represents a mainstay in the treatment of this increasingly prevalent arrhythmia. Prospective clinical trials investigating the efficacy of CA may poorly represent real-world patient populations. However, many real-world clinical datasets possess missing data, which may impede their applicability in research. Thus, we sought to use ensemble modeling to address missing data and develop a model to estimate the probability of AF recurrence following CA.

Methods

We retrospectively analyzed clinical variables in 476 patients who underwent an initial CA of AF. Univariate and multivariate logistic regression was performed to determine those variables predictive of AF recurrence. A multivariate logistic model was created to estimate the probability of AF recurrence after CA. Missing data were addressed using ensemble modeling, and variable selection was performed using the aggregate of multiple models.

Results

After analysis, six variables remained in the model: AF during the post-procedural blanking period, coexistence of atrial flutter, end-stage renal disease, reduced left ventricular ejection fraction, prior failure of anti-arrhythmic drugs, and valvular heart disease. Predictive modeling was performed using these variables for 1000 randomly partitioned datasets (80% training, 20% testing) and 1000 random imputations for each partitioned dataset. The model predicted AF recurrence with an accuracy of 74.34 ± 3.99% (recall: 54.03 ± 8.15%; precision: 89.30 ± 4.21%; F1 score: 81.08 ± 3.65%).

Conclusion

We successfully identified six clinical variables that, when modeled, predicted AF recurrence following CA with a high degree of classification accuracy. Application of this model to patients undergoing CA of AF may help identify those at risk of post-procedural AF recurrence.

## Introduction

Catheter ablation (CA) represents an increasingly utilized interventional technique aimed at reducing or eliminating the frequency and duration of episodes of symptomatic atrial fibrillation (AF). While CA has been shown to reduce the burden of AF, it has not been demonstrated to reduce the risk of stroke or death [1]. Consequently, CA is typically recommended for patients experiencing symptomatic AF. Considering the costs and risks associated with CA of AF, enhanced predictive models of ablation success derived from modeling techniques represent an attractive tool to physicians and AF patients alike. While generally low risk, CA of AF has been associated with complication rates that are not insignificant [2]. Additionally, the catheter ablation vs antiarrhythmic drug therapy for atrial fibrillation (CABANA) study’s findings reiterated that AF recurs in roughly half of patients undergoing AF ablation at a five-year follow-up [3]. Arbelo et al. reported an overall complication rate of pulmonary vein isolation (PVI) procedures approaching 7.8%, although the rate of complication in the ablation arm of CABANA was lower [4]. Consequently, this balances the combination of AF ablation success rates and procedural risk factors into the shared decision-making process between a PVI procedural candidate and their cardiac electrophysiologist. 

Numerous studies have examined the relationship between various patient characteristics and the recurrence of atrial arrhythmias post PVI. Underlying cardiovascular disease, valvular heart disease, increased age, AF classification (i.e. persistent versus paroxysmal), left atrial dimension, and presence of obstructive sleep apnea are patient characteristics associated with post-PVI atrial arrhythmia recurrence [5-12]. Early, as well as late, recurrence of AF and post-ablation atrial arrhythmias have various consequences as well [13]. In addition to clinical characteristics and recurrence monitoring, an ablation technique and the use of antiarrhythmic drug therapy to supplement CA have been shown to serve as predictors of procedural success [14-17].

Traditional prospective clinical studies rely on consistent access to pre-defined patient characteristics when formulating a predictive model for procedural success and outcomes. In conventional clinical settings, however, patient data are often incomplete or missing during the review of population-level databases. If a patient’s clinical information is unidentifiable or unobtainable, these meaningful patient data are frequently considered incomplete and may go unconsidered. A potential remedy to this widespread challenge is performing imputation in an effort to reasonably predict missing data points, thus allowing partial patient information to remain relevant while permitting its utilization in drawing qualified conclusions [18,19]. We hypothesized that creating a predictive algorithm through the use of ensemble modeling techniques on common clinical variables, including imputed missing data, could accurately predict the recurrence of atrial arrhythmias following CA of AF in a real-world setting. This article was previously presented as a poster presentation at the 2019 AHA Scientific Sessions on November 17, 2019. It was previously posted to the Research Square preprint server (https://www.researchsquare.com/article/rs-1842312/v1?) on July 29, 2022.

## Materials and methods

Patient population

Participants included all patients (n=476) undergoing their first PVI ablation between June 2011 and December 2017 at a tertiary medical center, each of whom had at least one ECG or 24-hour Holter monitor performed after the 90-day blanking period, but prior to one-year post-ablation. In the sampled clinical population, patients were post-operatively followed by either their referring provider or clinical electrophysiologist. Management of recurrent symptomatic AF was either performed by the procedural electrophysiologist or the patient’s referring physician. Patients were excluded if they had undergone a previous MAZE procedure. One patient was excluded because she died during the one-year observation period, thus not fulfilling the one-year observation requirement. Data from these patients were collected and analyzed retrospectively after approval from the Institutional Review Board. 

Data-gathering procedure

Information used to determine the clinical variables' status was charted prior to the CA, except for AF during the blanking period and antiarrhythmic drug status post-ablation. A retrospective chart review was performed, and data were collected on the following variables: age at the time of ablation, sex, body mass index, AF type, method of CA energy delivery (e.g. cryoballoon vs. radiofrequency), moderate or worse valvular heart disease, moderate or worse left ventricular concentric hypertrophy, coronary artery disease, history of myocardial infarction, evidence of prior reduced left ventricular ejection fraction (LVEF), heart failure with preserved EF (HFpEF), hypertension, prior transient ischemic attack, prior failure of an antiarrhythmic drug, prior cardiac surgery, end-stage renal disease, coexistence of atrial flutter, antiarrhythmic drugs prescribed prior to ablation, antiarrhythmic drugs prescribed for at least one year following ablation, AF during post-procedural blanking period, and time since initial AF diagnosis. The LVEF, left atrial diameter (LAD), and left atrial volume index (LAVI) were also included for analysis only when an echocardiogram had been performed less than six months before the ablation. AF type was categorized as paroxysmal: AF episodes were intermittent and lasting less than one week; persistent: AF episodes lasting greater than one week, but less than one year; and long-standing persistent: AF episodes lasting greater than one year. Clinical success was defined as the absence of a documented atrial arrhythmia, following the 90-day blanking period of greater than 30 s at the end of 12 months following the ablation.

Model generation

The variables were compared between patients with arrhythmia recurrence and those who remained free of arrhythmia. For categorical variables, relative frequencies, odds ratios (OR) using a specified baseline category, 95% confidence intervals for the OR, and p-values computed using Fisher’s exact test are reported. For continuous variables, the mean, 95% confidence interval, and the p-value computed using a t-test are reported. All analyses were performed in R™ (Vienna, Austria) ver. 3.5.0. Statistical significance was assessed using a=0.05.

Next, a logistic regression model was developed to identify factors associated with the recurrence of arrhythmia. To avoid sampling artifacts in the variables where imputed data were utilized, N=1000 imputed datasets were generated, and the regression model was fit on all the imputed datasets. Using all available variables, the logistic regression model is fit with forward stepwise regression using forward variable selection to determine which variables maximize the ability of the model to correctly predict AF recurrence. Bagging was then used to combine the results from these ensemble methods to select the variables to estimate the probability of recurrence of atrial arrhythmia within 12 months of the procedure.

Model validation

To study the model’s strength in predicting the recurrence of atrial arrhythmia, the classification model is divided randomly into two groups: training data, consisting of 80% (n=380) of the patients to construct the model; and testing data, consisting of the remaining 20% (n=96) of the patients. Division of data into training and testing datasets was performed after random imputation. To avoid sampling artifacts, we considered B=1000 randomly imputed data sets. For each imputed data set, the samples were divided randomly into M = 1000 training and testing data sets. For the models, we used only the variables selected through the forward selection procedure (as described previously). After estimating the model coefficients using the training data, the probability of recurrence of atrial arrhythmia is predicted for the patients in the testing dataset. Patients with a predicted probability greater than 50% are classified as having a recurrence of atrial arrhythmia. Compared to the observed recurrence of atrial arrhythmia, the accuracy of the prediction is calculated for the dataset as the percentage of patients who are correctly classified. The accuracy of the ensemble models is combined using bagging, and the mean accuracy of the M=1000 training/testing datasets is recorded.

Data imputation

For variables with missing observations, the data were randomly imputed. The observed data were used to identify appropriate models for the imputation process. The variables with missing observations were assumed to be missing completely at random. To complete the dataset, the missing values were imputed using random samples drawn from a normal distribution, with mean and standard deviation computed from the observed samples. This method is preferred to replace the missing values with the mean to study the variability of the model accuracy.

## Results

**Figure 1:**
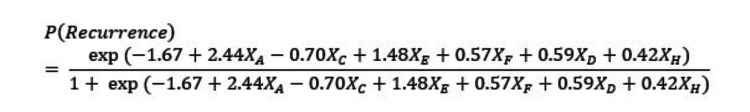
Model to Predict the Probability of Recurrence of Atrial Arrhythmia X_A_= AF documented during the blanking period; X_C_= Coexistence of atrial flutter; X_E_= End-stage renal disease; X_F_= Prior reduced left ventricular ejection fraction; X_D_= Prior failure of antiarrhythmic drugs; X_H_= Presence of valvular heart disease

Using the previously discussed method for model creation, six variables were selected that maximized the predictive value of the model. The addition of any other variables to the model did not increase the accuracy of outcome prediction. These variables were AF during the post-procedural blanking period, the coexistence of atrial flutter, end-stage renal disease, prior reduced LVEF, prior failure of anti-arrhythmic drugs, and moderate or worse valvular heart disease. The equation (Figure 1) seen below gives the estimated model to predict the probability of recurrence of atrial arrhythmia.

The total number of models that selected each of the variables is shown below (Table 1). In univariate analysis (Tables 2-3), valvular heart disease (p=0.0011), AF classification (p=0.0035), evidence of prior reduced EF (p=0.0124), remaining on antiarrhythmic drugs post-procedure for 12 months (p<0.0001), AF documented during the blanking period (p<0.0001), LAD (p=0.0125), months since initial AF diagnosis (p=0.0368), and LAVI (p=0.0492) all achieved statistical significance. In multivariate analysis, AF documented during the blanking period (p<0.0004) and valvular heart disease (p=0.0256) remained statistically significant individual predictors. Not all of the variables determined to be significant in univariate analysis strengthened the predictive value of the multivariate model. Coexistence of atrial flutter was the only variable included in the model that decreased the chance of AF recurrence. Of the 476 patients, 362 (76.05%) had at least one variable missing. The variables that required imputation were EF (n=12), LAD (n=256), LAVI (n=312), LV hypertrophy (n=17), and months since initial AF diagnosis (n=119). Approximately 41.8% of the patients included in this study had a recurrence of atrial arrhythmia during the observation period.

**Table 1:** Results of Multivariable Analysis ^a^The coefficients and standard error of the logistic model are reported for the complete data. The frequency of models in selecting the variable and significance is based on the 1,000 randomly imputed datasets.

Clinical variables	Coefficient (SE)^a^	Frequency of models selecting the variable	Frequency of significance
AF documented during the blanking period	2.44 (0.29)	1,000	1,000
Coexistence of atrial flutter	-0.70 (0.33)	963	2
End-stage renal disease	1.48 (0.75)	893	0
Prior reduced LVEF	0.57 (0.27)	893	190
Prior failure of anti-arrhythmic drugs	0.59 (0.28)	705	5
Moderate or worse valvular heart disease	0.42 (0.29)	1,000	906

**Table 2:** Results of Univariable Analysis: Significant Categorical Variables ^1^Confidence interval, ^2^Left ventricular ejection fraction

Clinical variables	Total number of patients in each category		No AF reoccurrence		AF recurrence		p-value	Odds ratio CI^1^
	#	(%)	#	(%)	#	(%)		
Valvular heart disease	n=476		n=277		n=199		0.001	[1.333,3.366]
No	371	78%	231	83%	140	70%		
Yes	105	22%	46	17%	59	30%		
Atrial fibrillation	n=476		n=277		n=199		0.004	
Paroxysmal	322	68%	203	73%	119	60%		
Persistent	134	28%	67	24%	67	34%		
Longstanding persistent	20	4%	7	3%	13	7%		
Evidence of prior reduced LVEF^2^	n=476		n=277		n=199		0.012	[1.098,2.586]
No	347	73%	214	77%	133	67%		
Yes	129	27%	63	23%	66	33%		
Antiarrhythmic drug post-procedure for 12 months	n=476		n=277		n=199		<0.0001	[1.549,3.405]
No	287	60%	190	69%	97	49%		
Yes	189	40%	87	31%	102	51%		
AF documented during the blanking period	n=476		n=277		n=199		<0.0001	[6.230,17.433]
No	344	72%	250	90%	94	47%		
Yes	132	28%	27	10%	105	53%		

**Table 3:** Results of Univariable Analysis: Significant Continuous Variables ^1 ^Standard deviation, ^2^ Confidence intervals, ^3^ Left atrial volume index

Continuous variables	No reoccurrence ± SD^1^	Reoccurrence ± SD^1^	CI of difference^2^	p-value
Left atrial diameter (cm)	4.09 ± 0.73	4.34 ± 0.74	[-0.455, -0.056]	0.013
Months since initial AF diagnosis (mo.)	33.37 ± 47.61	46.69 ± 66.1	[-25.759, -0.871]	0.037
LAVI^3 ^(mL/m^2^)	35.5 ± 13.5	39.81 ± 14.16	[-8.611, -0.016]	0.049

Using the previously discussed method for model validation, for the 1,000 randomly imputed datasets, the accuracy of the model is assessed. The model has a classification accuracy of 74.34%, with a standard deviation of 3.99%, when evaluated on the testing cohort. That is, the model correctly predicts the AF recurrence status of 74.34% of the patients in the testing cohort. The mean recall and precision averaged over the 1,000 randomly imputed datasets are 54.03 (8.15)% and 89.3 (4.21)%, respectively. The average F1 score is high - 81.08 (3.65%) - further emphasizing the accuracy of the model. When the analysis is performed using all of the variables, the accuracy of the model over 1,000 randomly imputed datasets is 71.05%, with a standard deviation of 4.21%. This indicates that the variables selected previously by the stepwise procedure are sufficient to achieve similar prediction accuracy in the model. An online tool for practitioners is made available at https://dnayyala.github.io/afib-recurrence-calc/.

## Discussion

We described the univariate and multivariate analysis of common clinical variables in predicting the recurrence of AF following PVI ablation using retrospective analysis of our institutional database. Many of the variables utilized in this study have been described in prior literature [20-24]. In our analysis, we demonstrated that it is possible to predict, with respectable reliability, the AF recurrence in post-CA AF patients, potentially better stratifying those patients who derive greater clinical benefit from PVI procedures. We provided the precision, recall, and F1 score to help better contextualize the accuracy of our model. To create a dataset that better reflects actual practice at a large, regional referral center, we did not exclude patients from our analysis owing to missing data. Rather, we sought to develop a model that accommodates missing data frequently encountered in real-world practice via the use of statistical imputation according to well-described techniques, to allow for the retention of as much data as possible [18]. The use of ensemble modeling techniques helps minimize the uncertainty associated with the imputation of missing data [25]. Furthermore, ensemble modeling creates a model with higher predictive accuracy compared to when just a single model is utilized. Imputation methods used in the creation of ensemble models also allow us to retain variables with missing values, which are otherwise discarded. 

The two variables that were found to be significant in univariate analysis and were selected for inclusion by the model (i.e. moderate or greater valvular heart disease and AF during the blanking period) are the variables that we are confident to play a significant role in predicting diminished success of PVI procedures. These variables have also been selected in prior large retrospective reviews of ablation registries [22-24]. Recurrent AF during the blanking period was also addressed by the adenosine following pulmonary vein isolation to target dormant conduction elimination (ADVICE) trial [23]. In this study, significant rates of atrial arrhythmia relapse were noted in patients experiencing documented AF during the post-CA blanking period. A study published in 2021 by Kim et al. found that 69.6% of the 751 patients who had a recurrence of AF in the first 90 days following CA also had late recurrence [26]. Another study by Yanagisawa et al. found that patients who underwent an early repeat CA following AF, documented during the post-CA blanking period, experienced significantly reduced AF recurrence rates when compared to those who did not undergo CA during the blanking period [27]. Additionally, a 2011 pilot study published by Pokushalov et al. showed a significant benefit in early CA for those with AF recurrence in the blanking period with AF initiated by atrial tachycardia, atrial flutter, or premature atrial beats [28]. Compared to deferring repeat CA until evidence of AF after the blanking period, a similar randomized control trial comparing early intervention in a high-risk subset of patients with early recurrence may be warranted. 

Intriguingly, the type of AF, whether paroxysmal or longstanding-persistent, was found to be significant in univariate analysis, as was seen in multiple prior studies. However, in multivariable analysis, the type of AF did not remain in the model [22,24]. This may indicate other factors associated with the type of AF may play a role in determining post-CA AF recurrence. Additionally, evidence exists that supports the role of and could be predicated upon the level of structural remodeling of the left atrium on post-CA AF recurrence [29]. It is more likely, however, that AF duration offers greater accuracy than the standard classification and thus may have more predictive value.

Study limitations

Our model was mainly limited in its creation owing to its retrospective nature and our single-center experience. Other inherent differences in ablation technique, pre- and peri-procedural management, and patient selection may result in some variability, although in our experience this was partially offset given the high-volume nature of our center’s AF ablation specialists. The outcome of CA is reported as a binary variable with clinical success being defined as the absence of atrial arrhythmia following CA outside of the blanking period. Defining the outcome of CA in terms of percentage reduction in AF burden might allow success to be determined on a more clinically relevant spectrum. Current guidelines state that patients who have failed anti-arrhythmic drug therapy for AF rhythm control have an indication for CA, thus limiting the clinical utility of this variable [30]. Another limitation we found was that the vast majority of the patient population is either Caucasian or African American, so the results of this study may be limited in its applicability to populations not encompassed by these two demographics.

## Conclusions

We identified six clinical variables that predicted AF recurrence following CA with a high degree of classification accuracy using ensemble modeling techniques and real-world clinical data from a large tertiary-referral center. We propose that this model may offer patients and physicians alike an additional tool when discussing and managing AF ablation procedural outcomes in conventional clinical practice. Further studies examining the prospective utility and accuracy of this model as applied to patients undergoing CA of AF may further clarify its applicability in more routine and widespread clinical use.
